# miR-124 downregulation leads to breast cancer progression via LncRNA-MALAT1 regulation and CDK4/E2F1 signal activation

**DOI:** 10.18632/oncotarget.7578

**Published:** 2016-02-22

**Authors:** Tongbao Feng, Fang Shao, Qiyong Wu, Xiaohang Zhang, Dongqin Xu, Keqing Qian, Yewen Xie, Shizhong Wang, Ning Xu, Yong Wang, Chunjian Qi

**Affiliations:** ^1^ Medical Research Center, The Affiliated Hospital of Nanjing Medical University, Changzhou No.2 People's Hospital, Changzhou, 213003, China; ^2^ Department of Oncology, The Affiliated Hospital of Nanjing Medical University, Changzhou No.2 People's Hospital, Changzhou, 213003, China; ^3^ Department of General Surgery, the Affiliated Hospital of Nanjing Medical University, Changzhou No.2 People's Hospital, Changzhou, 213003, China; ^4^ Section of Clinical Chemistry and Pharmacology, Department of Laboratory Medicine, Lund University, S-221 85 Lund, Sweden

**Keywords:** miR-124, MALAT1, cyclin-dependent kinase 4, cell cycle, breast cancer

## Abstract

The long non-coding RNA (lncRNA) metastasis-associated lung adenocarcinoma transcript 1 (MALAT1) has been recently shown to be dysregulated in several cancers. However, the mechanisms underlying the role of MALAT1 in breast cancer remain unclear. Herein, we showed that MALAT1 was aberrantly increased in breast cancer tissues and cells. MALAT1-siRNA inhibited breast cancer cell proliferation and cell cycle progression *in vitro* and *in vivo*. Furthermore, MALAT1 acted as an endogenous potent regulator by directly binding to miR-124 and down-regulating miR-124 expression. In addition, MALAT1 reversed the inhibitory effect of miR-124 on breast cancer proliferation and was involved in the cyclin-dependent kinase 4 (CDK4) expression. Taken together, our data highlight the pivotal role of MALAT1 in breast cancer tumorigenesis. Moreover, the present study elucidated the MALAT1-miR-124-CDK4/E2F1 signaling pathway in breast cancer, which might provide a new approach for tackling breast cancer.

## INTRODUCTION

Breast cancer is a malignant neoplasm originating from breast tissue and the most common cause of death of women throughout the world [1, 2]. Although many molecular triggers have been found to play a vital role in breast cancer development, many breast cancer patients fail to respond to initial chemotherapy [3]. Nevertheless, the underlying molecular mechanisms of breast cancer still remain unknown.

MicroRNAs play diverse roles in tumorigenesis [4–6] and in the progression of breast cancer [7, 8]. The microRNAs may act as oncogenes, tumor suppressors and modulators of tumor proliferation, invasion, apoptosis and therapy resistance [5, 7, 9–11]. An increasing body of evidence indicates that miR-124 is related to carcinogenesis. The miR-124 expression level is significantly suppressed in glioma [12], medulloblastoma [13, 14], oral squamous cell carcinoma (OSCC) [15], hepatocellular carcinoma (HCC) [16], bladder cancer [17] and breast cancer [18, 19]. Furthermore, the molecular mechanisms utilized by miR-124 to modulate the malignant phenotype of breast cancer cells are not fully understood. Cyclin-dependent kinase 4 (CDK4) is a master regulator of the cell cycle that belongs to the cyclin-dependent kinase family (CDK) [20]. CDK4 has been identified as the major oncogenic driver among the cell cycle components [20, 21]. Several tumor types, including leukemia [22], breast cancer [23] and lung cancers [24, 25], are dependent on cyclin D-dependent kinase activity, and we have previously identified CDK4 as a target of miR-124 [18].

Long noncoding RNAs (lncRNAs) are transcribed RNA molecules, exceeding 200 nucleotides in length but have no significant protein-coding potential [26]. LncRNAs can regulate gene expression [27] and participate in different biological processes, including the modulation of proliferation [28], migration [29, 30], and apoptosis [26, 31, 32]. In addition, lncRNAs can act as antisense transcripts or as decoys for splicing factors leading to splicing malfunctioning [33–35]. However, whether lncRNAs are involved in the regulation of breast cancer remains unclear.

Our present work aimed to identify the miRNAs and lncRNAs in breast cancer development. MicorRNA-124 was found to be a tumor suppressor in breast cancer. In searching for downstream targets of miR-124, we found that CDK4 was a direct target of miR-124 in breast cancer. We also found that MALAT1 may act as an endogenous potent regulator that represses miR-124 activity. MALAT1 was shown to increase CDK4 expression, which was also involved in the activation of the CDK4/E2F1 signaling pathway. Our results revealed a novel breast cancer regulation model that was comprised of the MALAT1-miR-124-CDK4/E2F1 pathway in breast cancer.

## RESULTS

### miR-124 aberrantly decreased in breast cancer and is associated with disease progression

**Figure 1:**
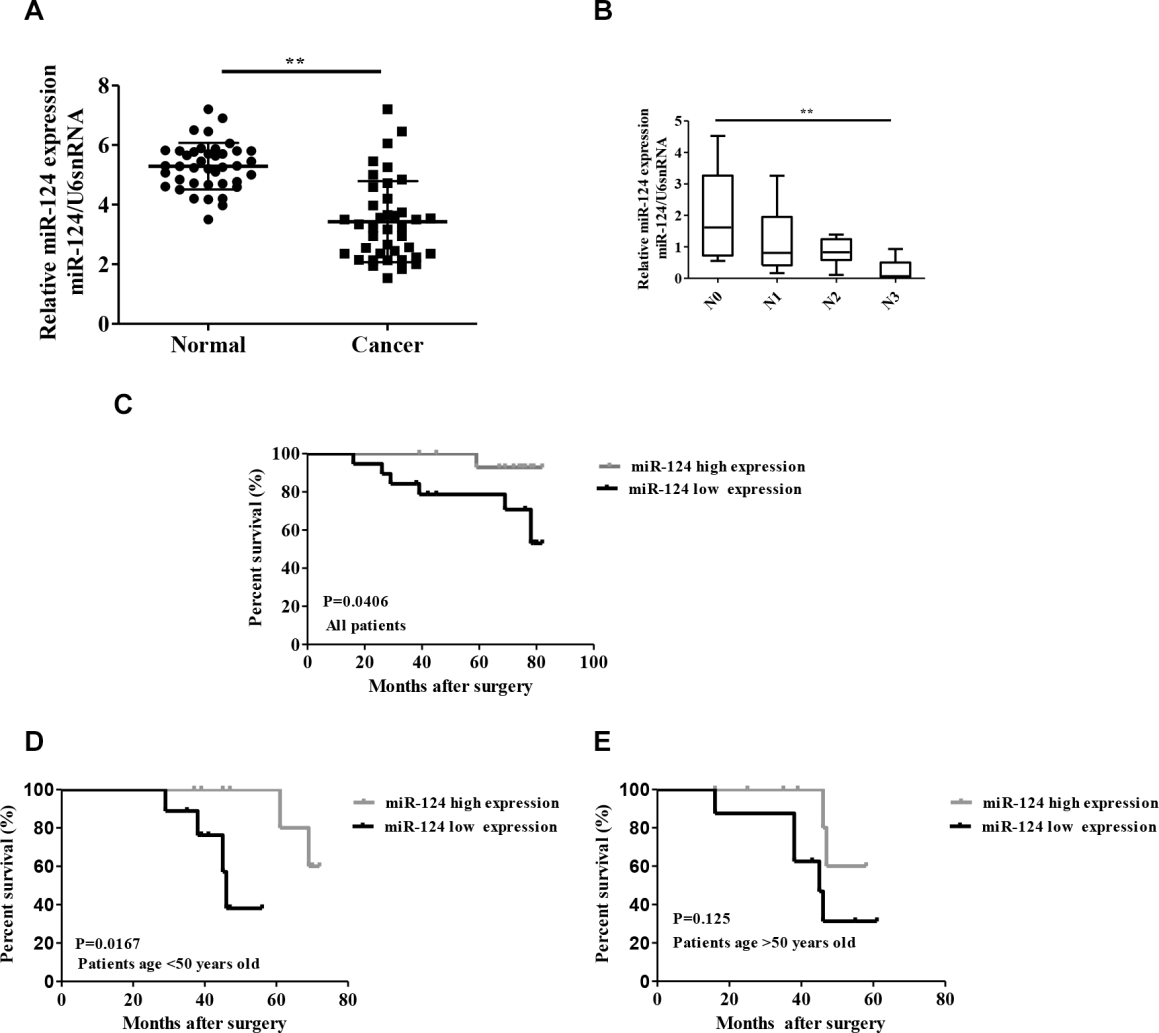
Relative miR-124 expression in breast cancer and its relationship with overall survival of breast cancer patients (**A**) miR-124 expression was examined by qRT-PCR and normalized to U6snRNA expression in breast cancer tissues (Cancer) compared with adjacent non-cancerous tissues (Normal). (**B**) miR-124 expression was examined by qRT-PCR in different advanced pathological stages N0, N1, N2 and N3. Kaplan-Meier analysis of overall survival for patients with breast cancer according to miR-124 expression. (**C**) All patients, (**D**) Patients with age younger than 50 years old, (**E**) Patients with age older than 50 years old. **p < 0.01, *p < 0.05.

To explore miR-124 levels in breast carcinogenesis, we used qRT-PCR to measure its expression in 40 pairs of breast cancer and adjacent non-cancerous tissues. The miR-124 level in cancer tissues was significantly decreased compared with those in adjacent tissues (*p* < 0.001, Figure 1A). In addition, clinic-pathological analysis showed that miR-124 expression was significantly correlated with the advanced pathological stages N0 and N3 (N0 and N1, *p = 0.140*; N0 and N2, *p = 0.1047*; N0 and N3, *p = 0.0045*, *p < 0.05*, Figure 1B). Supplementary Table S1 shows the clinicopathological differences between high and low miR-124 expression groups. miR-124 expression differed significantly according to age. High miR-124 expression was observed in patients younger than 50 years old (*p < 0.05*). No difference was found between miR-124 expression and other clinical features. To further investigate the correlation between miR-124 expression and breast cancer survival, Kaplan–Meier curves with log-rank analysis was performed. Overall survival was calculated as the time from the date of surgery resection to the date of last contact or death. Breast cancer patients with high miR-124 expression had a significantly longer survival time compared with low miR-124 expression (*P* = 0.0406, Figure 1C). Univariate analysis revealed that the expression of miR-124 (HR = 0.753, 95% CI: 0.388–1.460, *p = 0.0401, p < 0.05*) was associated with poor survival (Supplementary Table S2). In multivariate analysis, significant differences were identified between low- and high-miR-124 expression and patients with breast cancer (HR = 0.506, 95% CI: 0.201–1.274, *p = 0.0148*). Stratified analyses further revealed that in the age less than 50 group, patients with high miR-124 expression were significantly associated with better prognosis compared with those with low miR-124 expression (*p = 0.0167*, Figure 1D), however, no significant differences were observed between low- and high-miR-124 expression in patients older than 50 (*p = 0.125*, Figure 1E).

### miR-124 suppressed cell proliferation and induces G0/G1 cell cycle arrest in breast cancer

**Figure 2:**
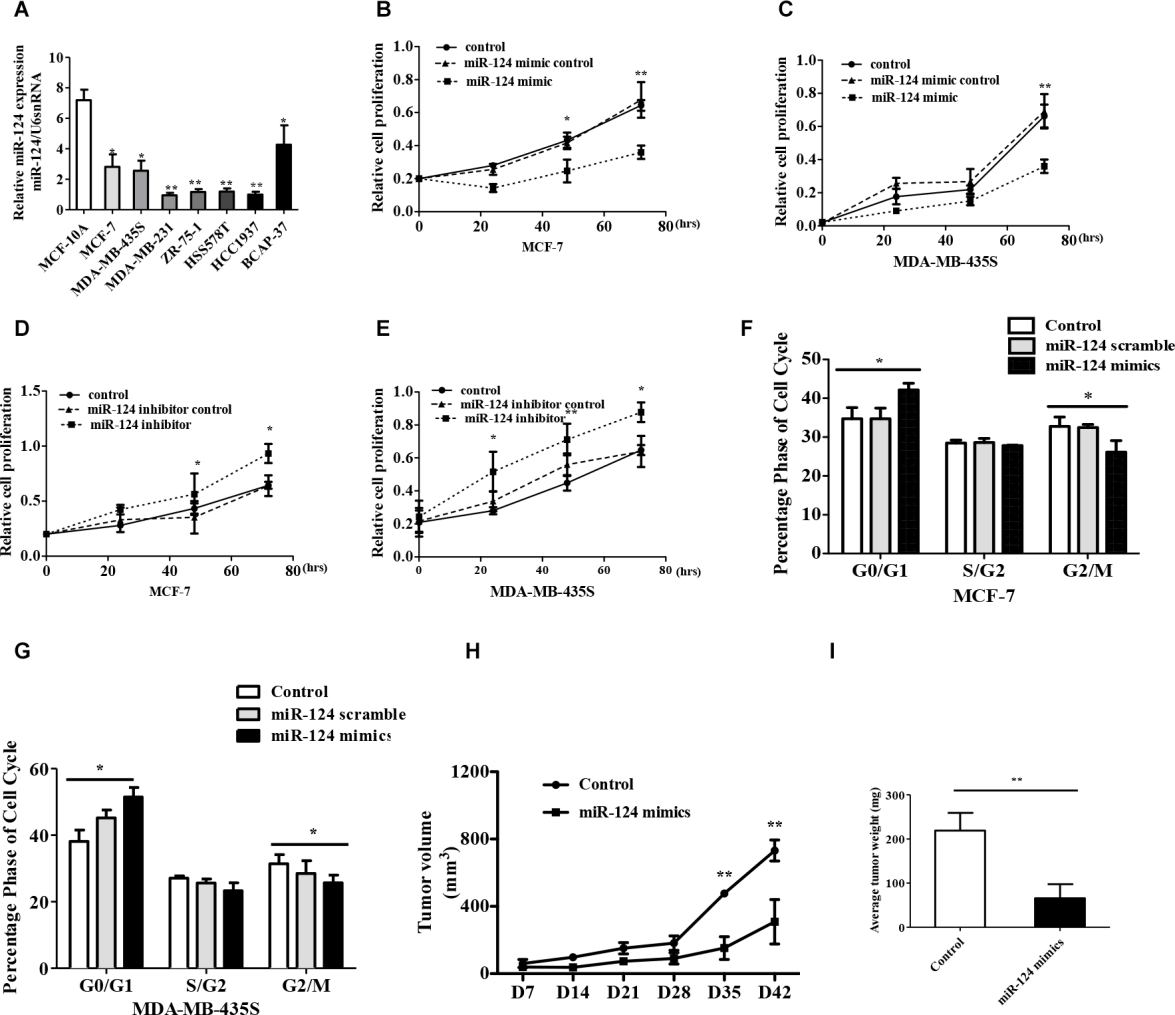
miR-124 inhibits breast cancer cell proliferation and induces cell cycle arrest *in vivo* and *vitro* (**A**) miR-124 expression levels were analyzed in different breast cell lines by qRT-PCR, and U6 snRNA was treated as internal control. (**B**, **C**) MTT detected miR-124 mimic effect on breast cancer cell proliferation. (**D**, **E**) MTT measured miR-124 inhibitor effect on breast cancer cell proliferation. Cell cycle profile was examined by flow cytometry with propidium iodide staining, and the cell number was counted according to DNA content of G0/G1, S and G2/M phases. (**F**) MCF-7, (**G**) MDA-MB-435S. (**H**) Tumor growth curves measured after injection of MCF-7 cells stably transfected with miR-124 mimic or control. The tumor volume was calculated every 7 days. (**I**) The tumor mass was determined when the mice was sacrificed. The data are shown as the mean ± SD from three independent experiments with similar results. *p < 0.05, **p < 0.01.

Based on the above observations, miR-124 expression analysis was conducted among 7 different breast cancer cell lines (MCF-7, MDA-MB-435S, MDA-MB-231, ZR-75-1, HSS578T, HCC1937 and BCAP-37) and normal human mammary gland epithelial cell line (MCF-10A). We noted that miR-124 was obviously inhibited in 7 breast cancer cell lines compared with the MCF-10A cells (Figure 2A). Then, we transfected MCF-7 cells with a miR-124 mimic or inhibitor (Supplementary Figure S1). As expected, transfection of the miR-124 mimic decreased the proliferation of the MCF-7 and MDA-MB-435S breast cancer cell lines compared with cells transfected with miR-124 mimic control (Figure 2B and 2C). In contrast, transfection of the miR-124 inhibitor significantly increased the proliferation of the breast cancer cell lines MCF-7 and MDA-MB-435S compared with cells transfected with the miR-124 inhibitor control (Figure 2D and 2E).

We further analyzed cell cycle distribution using flow cytometry in miR-124-treated MCF-7and MDA-MB-435S cells (Figure 2F and 2E). The data showed that after transfection with the miR-124 mimic, the percentage of cells in the G0/G1 phase increased from 34.8 to 51.49% (*p < 0.05*). These results demonstrated that G1-S cell cycle progression was inhibited following the overexpression of miR-124 in breast cancer cell lines.

To determine the effect of miR-124 on tumor growth *in vivo*, we used xenograft model in which the MCF-7 cells treated with miR-124 mimic or control were subcutaneously injected into nude mice. No animal death was observed during the course of the treatment, and no other complications such as skin necrosis were detected due to infection. After 42 days, we observed slower tumor growth in the miR-124 mimic group compared with the control group (Figure 2H). The average weight of the tumors developed from miR-124 mimic-transfected MCF-7 cells significantly decreased compared with the tumor mass in the control group (Figure 2I). These results suggest that miR-124 overexpression can inhibit the proliferation capacity of breast cancer cells *in vivo*.

### MALAT1 inhibited miR-124 expression in breast cancer

**Figure 3:**
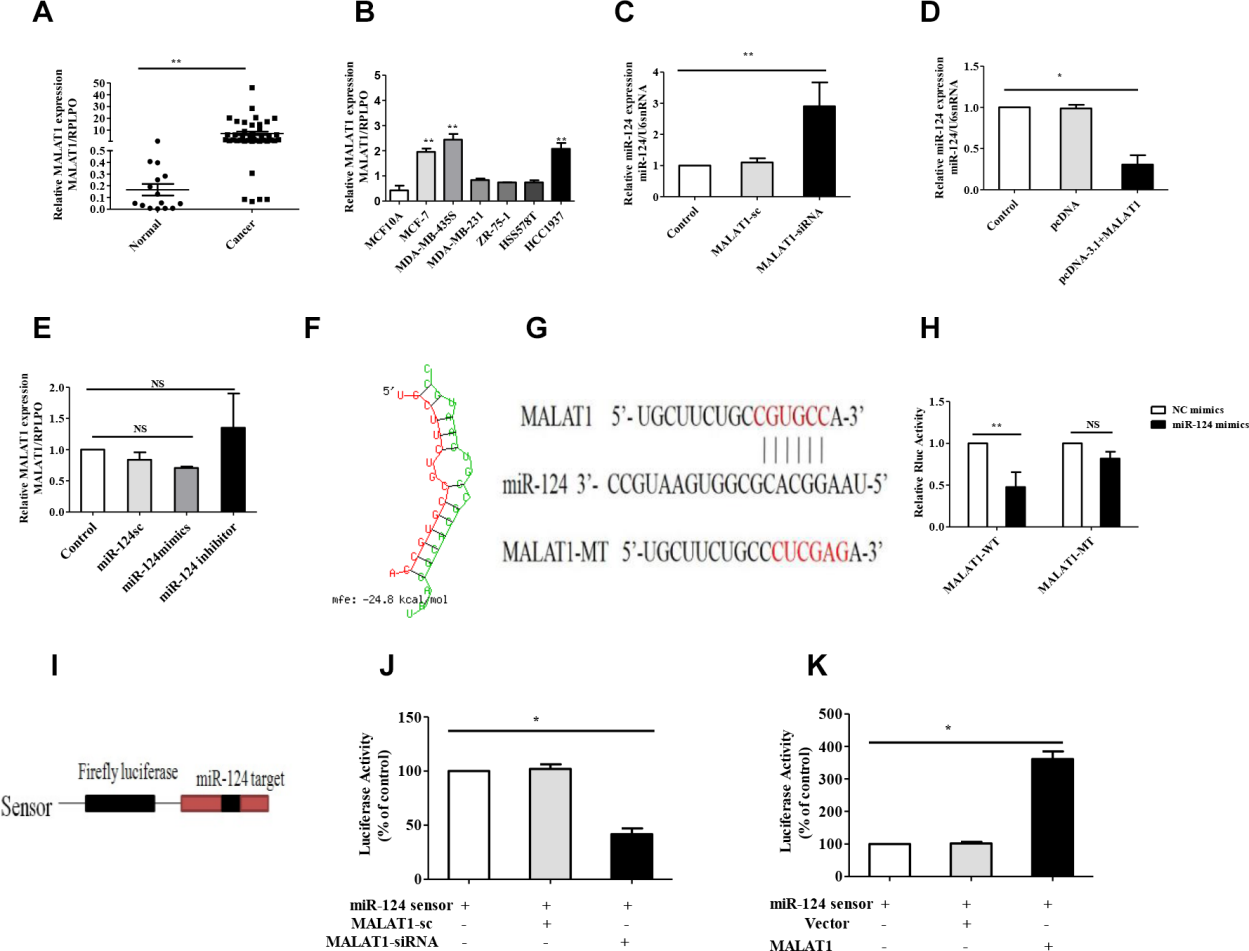
MALAT1 reduces miR-124 expression in breast cancer (**A**) MALAT1 expression was examined by qRT-PCR and normalized to RPLPO expression in breast cancer tissues (Cancer) compared with adjacent non-cancerous tissues (Normal). (**B**) MALAT1 expression levels were analyzed in different breast cancer cell lines by qRT-PCR and RPLPO was treated as an internal control. (**C**, **D**) miR-124 expression levels were analyzed in different breast cell lines with siMALAT1 and pcDNA-MALAT1 treated by qRT-PCR, and U6snRNA was treated as an internal control. (**E**) MALAT1 expression levels were analyzed in breast cancer cells treated with the miR-124 mimic, miR-124 inhibitor and miR-124 scramble by qRT-PCR, and RPLPO was treated as the internal control. (**F**) Predicted target sites with a 6-nt seed match for MALAT1 and miR-124.Red, MALAT1; Green, miR-124. (**G**) miR-124 target sequence on MALAT1 and its mutant sequence. The red letters show the mutated site and the seed region. (**H**) The relative luciferase activities of luciferase reporters containing a Malat1 fragment with the miR-124 target site (Luc- MALAT1-wt) or mutation site (Luc-MALAT1-mut) were detected 24 hours after co-transfection with the miR-124 or the scramble NC mimics. (**I**) miR-124 sensor construct. Human genomic sequences (400 bp) flanking pre-miR-124 were reverse-inserted into the downstream of luciferase gene in pGL3 vector. (**J**) Breast cancer cells were treated with CHRF-siRNA and its scramble and pcDNA-MALAT1 (**K**), then transfected with miR-124 sensor. Luciferase activity was analyzed. The data are shown as the mean ± SD from three independent experiments. *p < 0.05, **p < 0.01, *NS*, No significant difference.

Recent studies have suggested that lncRNAs contain motifs with sequences complementary to miRNAs; therefore, lncRNAs may act as an endogenous RNA regulator that interacts with miRNAs and influences miRNA expression. Previous studies have demonstrated that MALAT1 expression is increased in multiple cancers, and our results supported these findings as it was significantly increased in breast cancer tissues compared with the adjacent non-tumor tissues (Figure 3A). We also found that MALAT1 was increased in most breast cancer cells (Figure 3B). As mentioned above, miR-124 was decreased in breast cancer tissues (Figure 1A) and breast cancer cells (Figure 2B). Therefore, MALAT1 upregulation and miR-124 down-regulation is a frequent event in breast cancer tissues (Supplementary Figure S2A) and breast cancer cells (Supplementary Figure S2B), and may be involved in malignant tumor development. To further validate the negative regulation of MALAT1 on miR-124, we silenced MALAT1 by using its specific siRNAs (siMALAT1) and induced MALAT1 by constructing a MALAT1 overexpression vector. MALAT1 was downregulated by siMALAT1 (Supplementary Figure S2C), and MALAT1 was upregulated by pcDNA-MALAT1 vector (Supplementary Figure S2D). The siMALAT1 was applied in the following experiments, and dramatically enhanced miR-124 expression was observed in MCF-7 cells (Figure 3C). In contrast, miR-124 was downregulated in the MCF-7 cells with MALAT1 overexpression (Figure 3D). We found that MALAT1 expression had no effect in breast cancer cells treated with the miR-124 mimic or miR-124 inhibitor (Figure 3E). In contrast, Liu et al., have reported that enforced miR-124 expression in cervical cancer cells reduced MALAT1 levels [36]. We also found that MALAT1 and miR-124 expression were decreased in breast cancer cells treated with siAGO (RNA- induced silencing complex, RISC, core family number protein) (Supplementary Figure S2E). These data show that MALAT1 inhibited miR-124 expression in breast cancer cells.

To understand the mechanism by which MALAT1 inhibited miR-124 levels, we investigated whether MALAT1 interacts with miR-124. We compared the MALAT1 sequence with miR-124 using RNAhybrid and noticed that MALAT1 contains a miR-124 target site (Figure 3F and 3G). We produced a luciferase construct of MALAT1 RNA (Luc- MALAT1-wt) and a mutated form (Luc- MALAT1-mut). The luciferase assay suggested that miR-124 inhibits the luciferase activity of MALAT1 RNA, but had a lesser effect on the mutated form of MALAT1 RNA compared with the wild type (Figure 3H). These results reveal that MALAT1 may interact with miR-124 by this putative binding site. To understand whether MALAT1 affects miR-124 activity, we constructed a miR-124 sensor (Figure 3I). The miR-124 sensor construct contains a perfect miR-124 target, and a reduced luciferase activity of the sensor indicates the induction of miR-124 activity. Our results showed that the luciferase activity of the miR-124 sensor was decreased in cells treated with siMALAT1 (Figure 3J), suggesting the induction of miR-124 activity. Enforced expression of MALAT1 induced a reduction in miR-124 activity (Figure 3K). Taken together, our results reveal that MALAT1 may act as an endogenous potent regulator that reduces miR-124 expression.

### MALAT1 reverses the inhibitory effect of miR-124 on cell proliferation and the cell cycle in breast cancer

**Figure 4:**
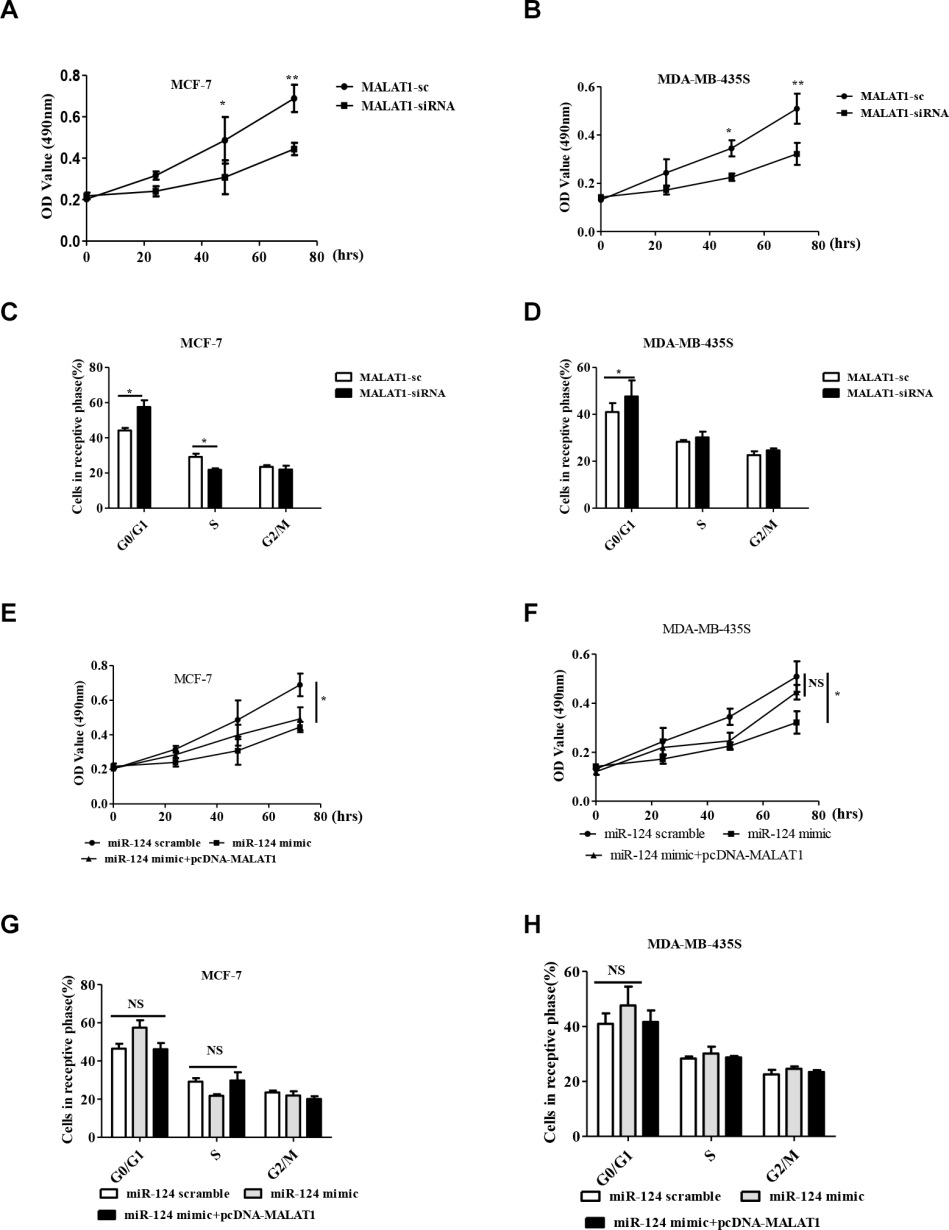
MALAT1 reverses the inhibitory effect of miR-124 on cell proliferation and cell cycle in breast cancer cells *in vitro* (**A**, **B**) Cell proliferation was assayed in MALAT1-sc- and MALAT1-siRNA- transfected MCF-7 and MDA-MB-453S cells by MTT at 0 h, 24 h, 48 h and 72 h. (**C**, **G**) MCF-7. (**D**, **H**) MDA-MB-435S. Cell cycle profile was examined by flow cytometry with propidium iodide staining, and the cell number was counted according to DNA content of G0/G1, S and G2/M phases. (**E**, **F**) Cell proliferation was assayed in miR-124 scramble, miR-124 mimic and miR-124 mimic + pcDNA-MALAT1 transfected MCF-7 and MDA-MB-453S cells by MTT at 0 h, 24 h, 48 h and 72 h. The data are shown as the mean ± SD from three independent experiments. *p < 0.05, **p < 0.01, *NS*, No significant difference.

Base on the above, inhibitory effect of MALAT1 on miR-124 in breast cancer, we thus tested whether MALAT1 involved in cell proliferation and the cell cycle. Compared with MALAT1-sc treatment groups, MALAT1-siRNA significantly inhibited cell proliferation at 48 and 72 h after transfection into MCF-7 and MDA-MB-435S cells (Figure 4A and 4B). We further analyzed the cell cycle distribution using flow cytometry in MALAT1-siRNA treated MCF-7 and MDA-MB-435S cells. The data showed that after transfection with the MALAT1-siRNA, the percentage of cells in the G0/G1 phase increased from 44.2% to 55.7% (*p < 0.05*), compared with the MALAT1-sc treatment groups (Figure 4C and 4D). Our data shows that MALAT1-siRNA inhibits cell proliferation and induces G0/G1 cell cycle arrest in breast cancer cells. Further, we used miR-124 scramble, miR-124 mimic and miR-124 mimic+pcDNA-MALAT1 to transfect MCF-7 and MDA-MB-435S cells. The data showed that miR-124 mimic+pcDNA-MALAT1 significantly inversed the inhibitory effect of the miR-124 mimic on MDA-MB-435S cells proliferation and partially inverses the inhibitory effect of the miR-124 mimic on MCF-7 cell proliferation (Figure 4E and 4F). Furthermore, miR-124 mimic+pcDNA-MALAT1 inversed the inhibitory effect of the miR-124 mimic on the cell cycle in both cells (Figure 4G and 4H).

### MALAT1 suppressed tumor growth through miR-124 *in vivo*

**Figure 5:**
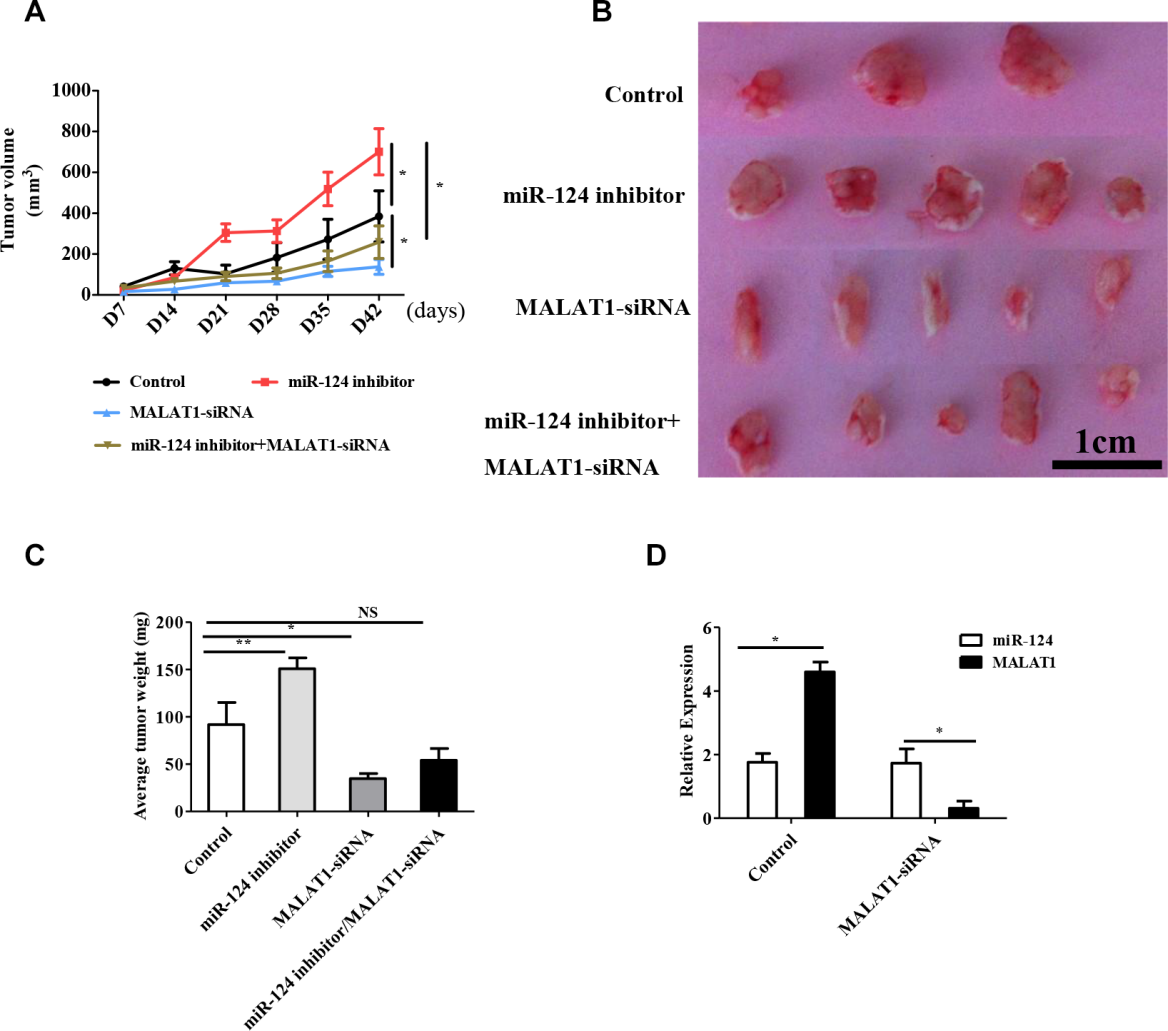
MALAT1 regulates tumor growth through miR-124 *in vivo* (**A**)Tumor growth curves measured after injection of MCF-7 cells stably transfected with MALAT1-siRNA, miR-124 inhibitor, miR-124 inhibitor+MALAT1-siRNA and PBS as the control. The tumor volume was calculated every 7 days. (**B**, **C**) The tumor mass was determined when the mice were sacrificed. (**D**) qRT-PCR detected miR-124 and MALAT1 expression in tumor tissues from the control group and MALAT1-siRNA group. The data are shown as the mean ± SD from MALAT1-siRNA (*n* = 5), miR-124 inhibitor (*n* = 5), miR-124 inhibitor+MALAT1-siRNA (*n* = 5) and PBS as the control (*n* = 3) from three independent experiments with similar results. *p < 0.05, **p < 0.01, *NS*, No significant difference.

To further validate the *in vivo* significance of MALAT1 on tumor growth *in vivo*, we used a xenograft model in which the MCF-7 cells treated with MALAT1-siRNA (5/mice), miR-124 inhibitor (5/mice), miR-124 inhibitor+MALAT1-siRNA (5/mice) and PBS as control (3/mice) were subcutaneously injected into nude mice. During the entire tumor growth period, we observed slower tumor growth in the MALAT1-siRNA group compared with the control group. We also observed faster tumor growth in the miR-124 inhibitor group compared with the control group and the miR-124 inhibitor+MALAT1-siRNA inversed the effect of the miR-124 inhibitor on tumor growth *in vivo* (Figure 5A and 5B). We also observed that the average weight of tumors from the miR-124 inhibitor group was significantly higher than the tumors in the control group. In addition, the tumor mass from the MALAT1-siRNA group was significantly smaller than control group, and the tumor from the miR-124 inhibitor+MALAT1-siRNA showed no differences compared with the control group (Figure 5C). As shown in Figure 5D, the miR-124 and MALAT1 expression levels were negative in control group and in MALAT1-siRNA group. In addition, Hatziapostolou et al., have reported that the systemic delivery of miR-NC or miR-124 did not affect liver and kidney function and did not have any toxicity effects on essential organs [16]. In our research, we found that the miR-124 inhibitor could affect the average weight of the spleen (Supplementary Figure S3A), liver (Supplementary Figure S3B) and lung (Supplementary Figure S3C), and that MALAT1 also inverted these effect. Importantly, the mechanism of how MALAT1 and miR-124 affected the weight of the spleen, liver and lung needed further study. Taken these together, we conclude that MALAT1 inverts the inhibitory effect of miR-124 on the tumor growth of breast cancer cells *in vitro* and *in vivo*.

### MALAT1 increased the expression of CDK4, a target of miR-124

In previous study, we found that Cyclin-dependent kinase 4 (CDK4) is a direct target of miR-124 and that miR-124 inhibited cell proliferation via CDK4 in breast cancer [18]. Thus, we investigated the expression of MALAT1, CDK4 and miR-124 in breast cancer tissues. We observed that the expression levels of MALAT1were positively associated with CDK4 (Supplementary Figure S4A, *R* = 0.611, *p < 0.05*). Moreover, MALAT1 expression levels were inversely associated with miR-124 (Supplementary Figure S4B, *R* = −0.5363, *p < 0.05*).

**Figure 6:**
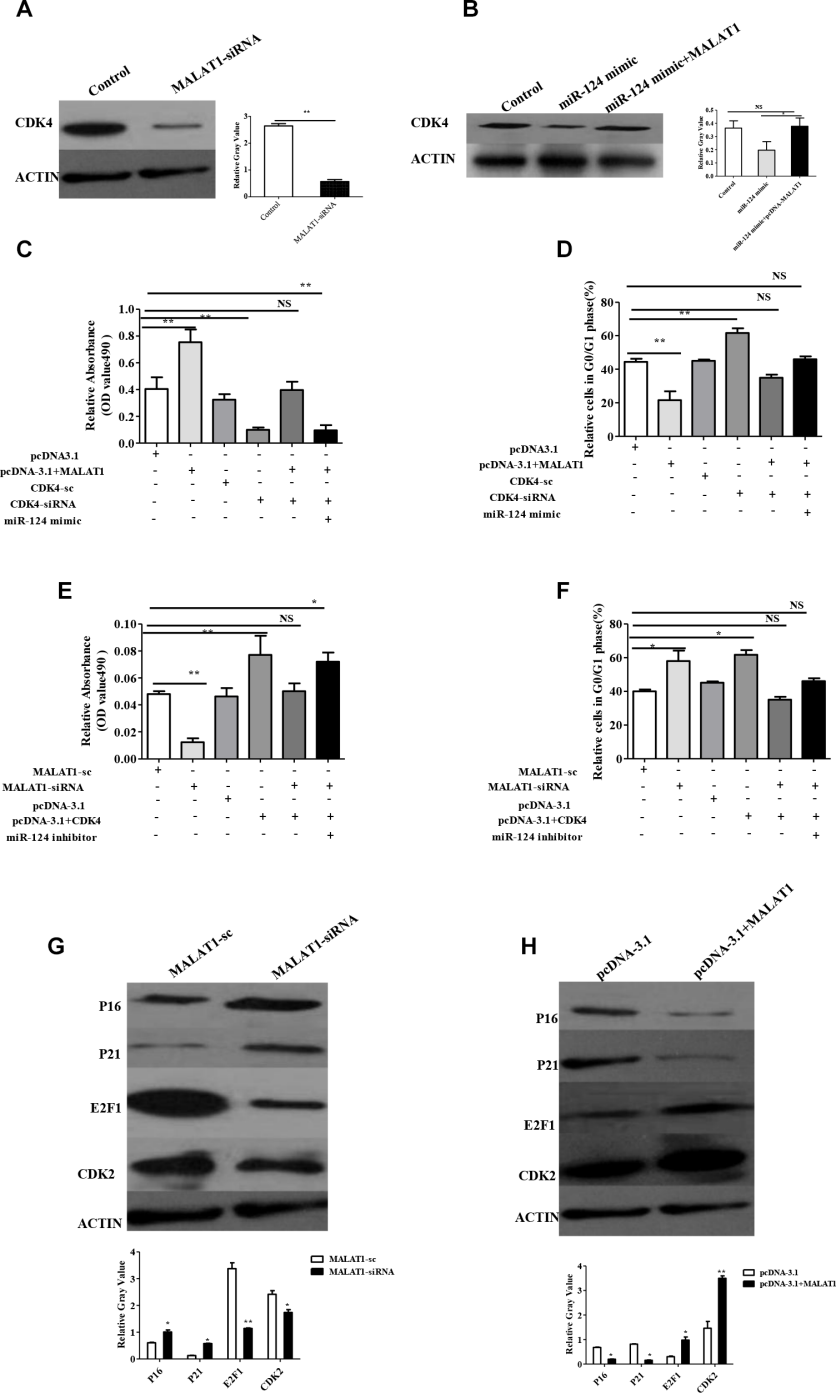
MALAT1 regulates the expression of CDK4, target of miR-124, also through CDK4/E2F1 signaling pathway in breast cancer (**A**, **B**) The expression of CDK4 was determined by western blot. MALAT1-sc and miR-124 scramble as control. The relative protein expression levels were obtained from three independent experiments. (**C**) The effect of MALAT1, CDK4-siRNA and miR-124 mimic on breast cancer cell proliferation measured by MTT. (**D**) The effect of MALAT1, CDK4-siRNA and miR-124 mimic on cell cycle G0/G1 phase in breast cancer. (**E**)The effect of MALAT1-siRNA, CDK4 and miR-124 inhibitor on breast cancer cell proliferation measured by MTT. (**F**) The effect of MALAT1-siRNA, CDK4 and miR-124 inhibitor on cell cycle G0/G1 phase in breast cancer. The data are shown as the mean ± SD from three independent experiments. (**G**) CDK2, E2F1, P21, P16 were determined by western blot from MALAT1-sc and MALAT1-siRNA treated breast cancer cells. (**H**) CDK2, E2F1, P21, P16 were determined by western blot from pcDNA-3.1 and pcDNA-3.1+MALAT1 treated breast cancer cells. The data are shown as the mean ± SD from three independent experiments, β-ACTIIN was used as a control *p < 0.05, **p < 0.01.

We have also shown that miR-124 expression levels were inversely associated with CDK4 expression (Supplementary Figure S4C). Furthermore we wanted to know whether MALAT1 increased CDK4 expression in breast cancer. The data showed that CDK4 was inhibited at both mRNA and protein levels in breast cancer cells treated with MALAT1-siRNA (Supplementary Figure S4D) compared with MALAT1-sc (Figure 6A). Our previous studies have reported that miR-124 inhibited CDK4 expression in breast cancer cells, therefore, we testes whether MALAT1 inverted the inhibitory effect of miR-124 on CDK4 expression. The CDK4 mRNA and protein levels were inverted in miR-124 mimic +pcDNA-MALAT1 group compared with the miR-124 mimic alone group as shown in Figure 6B and Supplementary Figure S4E. We further investigated whether MALAT1 increased breast cancer cell proliferation and the cell cycle by targeting CDK4. The results showed that MALAT1 overexpression accelerated breast cancer cells proliferation, whereas CDK4-siRNA inhibited cancer cell proliferation. In addition, we validated that the treatment with MALAT1 and CDK4-siRNA attenuated the effect of MALAT1 on breast cancer cells proliferation. However, the miR-124 mimic revised the overexpression of MALAT1-induced proliferation in breast cancer cells (Figure 6C). We also found that the overexpression of MALAT1 promoted the cell cycle G0/G1 phase, and the miR-124 mimic revised this event (Figure 6D). The results also showed that MALAT1-siRNA repressed breast cancer cell proliferation, whereas CDK4 overexpression induced breast cancer cell proliferation. We validated that the treatment with MALAT1-siRNA and CDK4 attenuated the effect of MALAT1 on breast cancer cells proliferation. However, the miR-124 inhibitor rescued MALAT1-siRNA-respressed proliferation in breast cancer cells (Figure 6E). In addition, we found that MALAT1-siRNA arrested the cells in the cell cycle G0/G1 phase, and the miR-124 inhibitor rescued this event (Figure 6F). Taken together, these data show that MALAT1 is positively associated with CDK4 and negatively associated with miR-124 in clinical breast cancer tissues, which increased CDK4 expression though miR-124 in breast cancer cells.

### MALAT1 is involved in the CDK4/E2F1 signaling pathway in breast cancer cells

CDK4 is a master regulator of the cell cycle in the G1-S phase checkpoint and has been identified as the major oncogenic driver in cell cycle. E2F1 is the main target of CDK4 and promotes cell proliferation. Our results showed that CDK4 was induced by MALAT1 expression, therefore, we wondered whether CDK4/E2F1 signaling is involved in this event. We found that E2F1 and CDK2 were downregulated, and E2F2 was not altered, while p16 and p21 were induced in MALAT1-siRNA treated cells compared with MALAT1-sc (Figure 6G). On the other hand, we also found that E2F1and CDK2 were promoted, while p16 and p21 were suppressed in pcDNA-MALAT1 treated compared with pcDNA- alone (Figure 6F). In addition, we also found that MALAT1 increased these molecules in RNA level (Supplementary Figure S5A and S5B). The data suggest that MALAT activates the CDK4/E2F1 signaling pathway in breast cancer. We found that miR-124 regulated the expression of E2F1, P21, p27, which could be inverted by MALAT1 in breast cancer cells; however, miR-124 has no effect on the expression of E2F2 and CDK1 (Supplementary Figure S5C). Taken together, miR-124 is suppressed by MALAT1, and involved in cell proliferation and the cell cycle via the CDK4/E2F1 signaling pathway in breast cancer.

## DISCUSSION

Studies have shown that miRNAs act as tumor suppressors or oncogenes in cancer [7, 9, 11, 27]. To gain a better understanding of the roles of miR-124 in modulating breast cancer cells, we first measured the relationship between miR-124 expression and the overall survival of breast cancer patients. We found that breast cancer patients with high miR-124 expression had a significantly longer survival time compared with the patients with low miR-124 expression. In our previous studies, we found that miR-124 inhibited cell proliferation by targeting CDK4 in breast cancer [18], however, the mechanism of miR-124 expression remains to be elucidated.

Long non-coding RNAs (lncRNAs) are defined as endogenous cellular RNAs more than 200 nucleotides in length that lack an open reading frame of significant length, which can regulate protein-coding genes at epigenetic, transcriptional, and post-transcriptional levels and play central roles in physiological processes [37, 38]. In our present work, we have found that MALAT1 expression was significantly upregulated in breast cancer tissues compared with the adjacent non-tumor tissues and breast cancer cells, MALAT1-siRNA significantly inhibited cell proliferation after transfection into MCF-7 and MDA-MB-435S cells. Recent studies reported that MALAT1 was involved in tumors progression [32, 39, 40]. In this study, we found that MALAT1 suppressed miR-124 expression in breast cancer, which inverted the inhibitory effect of miR-124 on the tumor growth of breast cancer cells *in vitro* and *in vivo*. Liu et al., reported MALAT1-miR-124-RBG2 axis is involved in the growth and invasion of HR-HPV-positive cervical cancer cells [36]. Although, Liu et al., reported that enforced miR-124 expression in cervical cancer cells reduced MALAT1 levels, we found that MALAT1 expression could be regulated in breast cancer cells treated with miR-124 mimic or miR-124 inhibitor. MALAT1 controls cell cycle progression by regulating the expression of oncogenic transcription factor B-MYB [39]. Jiang et al. found that MALAT1 regulates the cell cycle regulation molecules cyclinD1 and CDK6 in cervical cancer [41]. In the present study, we found that CDK4, E2F1 and CDK2 were decreased, and E2F2 was not altered, while p16 and p21 were increased in MALAT1-siRNA treated compared with MALAT1-sc cells. We also found that CDK4, E2F1, CDK2 were promoted, while p16 and p21 were suppressed in pcDNA-MALAT1 treated cells. The data suggested that MALAT1 was involved in cell proliferation through the CDK4/E2F1 signaling pathway in breast cancer.

In summary, our data showed that miR-124 inhibited cell proliferation by CDK4 and MALAT1 induced cell proliferation by decreasing miR-124 in breast cancer. In addition, MALAT1 was involved in CDK4/E2F1 signaling pathway to increase cell proliferation. Thus, our findings provided new insights into the mechanism of breast cancer cell proliferation modulated by MALAT1-miR-124 -CDK4/E2F1 signaling pathway in the development of breast cancer.

## MATERIALS AND METHODS

### Tissue specimens

Breast cancer and adjacent normal tissue samples were obtained with informed consent from patients who had undergone breast cancer surgery at the Affiliated Hospital of Nanjing Medical University, Changzhou No. 2 People's Hospital, Changzhou, China. The clinic-pathological features are shown in Supplementary Table S1. Tumor and corresponding non- tumor fresh specimens were snap-frozen in liquid nitrogen and stored at −80°C immediately after resection for the extraction of RNA and protein. This study was approved by the Research Ethics Committee of Nanjing Medical University.

### Cell lines and cell culture

Human breast cancer cell lines (MCF-7, Bcap-37 and MDAMB-435S) were obtained from the Central Lab of the Affiliated Hospital of Nanjing Medical University, Changzhou No. 2 People's Hospital. HCC1937, ZR-75-1, HS578T and MDA-MB-231 were purchased from the Institute of Biochemistry and Cell Biology of the Chinese Academy of Sciences (Shanghai, China). The cell lines were cultured in RPMIMedium1640 and Dulbecco's Modified Eagle's Medium (DMEM) (GIBCO, Invitrogen) containing 10% fetal bovine serum (FBS, Invitrogen) and were grown in a humidified 5% CO_2_ incubator at 37°C.

### RNA extraction and expression analysis

Total RNA was extracted using TRIzol reagent (Invitrogen). Reverse transcription was performed as previously described [18]. Primers are listed in Supplementary Table S3. U6snRNA, GAPDH and ACTIN were used as endogenous controls.

### Western blot

Western blot analysis to assess protein expression was performed as previously described [18]. The antibodies against E2F1, E2F2 and β-ACTIN were purchased from Cell Signaling, and anti-CDK2, -CDK4, -P16, and -P21 antibodies were purchased from Proteintech. The cells were washed in phosphate-buffered saline (PBS), and proteins was extracted in RIPA buffer. Lysates were cleared by centrifugation, and protein concentrations were estimated using the Bio-Rad protein assay (Bio-Rad, Milan, Italy). Then, 50 μg of protein/lane was loaded onto an acrylamide gel and separated by SDS–PAGE under denaturing conditions. The separated proteins were then transferred electrophoretically to a polyvinylidene fluoride (PVDF) membrane soaked in transfer buffer. Non-specific binding was blocked by incubating the blots in 5% non-fat dry milk in PBST for 60 min. After washing, the blots were incubated overnight at 4°C with the primary antibody and β-ACTIN was used as a reference protein. After incubation with the primary antibodies and washing in PBST, anti-mouse or anti-rabbit secondary antibody (both diluted 1:5000) was added (as appropriate) and incubated for 2 h at room temperature. In each experiment, the same amount of protein was used, and each experiment was repeated independently at least three times.

### RNA oligoribonucleotides and cell transfections

Both miR-124 and all RNA oligoribonucleotides for *in vitro* studies were purchased from Genepharma (Shanghai, China). The small interfering RNAs (siRNAs) specifically target human MALAT1, CDK4, AGO and the negative control RNA duplex. Their sequences were listed in Supplementary Table S3. The transfection of RNA oligoribonucleotides was performed using Lipofectamine 2000 (Invitrogen). Unless otherwise indicated, 100 nM of RNA duplex or 80nM of miRNA inhibitor were used for each transfection, and all of the experiments were repeated in triplicate.

### Bioinformatics analyses

The online bioinformatics programs, miRanda (http://www.microrna.org), Targetscan (http://www.targetscan.org) and RNAhybrid (http://bibiserv.techfak.uni-bielefeld.de/rnahybrid/) were applied to predict the target site of miR-124 and MALAT1.

### Plasmid generation

The MALAT1 sequence was synthesized and subcloned into the pcDNA3.1 (Invitrogen, Shanghai, China) vector. Ectopic expression of MALAT1 was achieved via pcDNA-MALAT1 transfection, with an empty pCDNA3.1vector used as a control.

### Dual-luciferase assay

Cells grown in the 96-well plate were co-transfected with either empty vector or miR-124 and luciferase reporter comprising either the wild type or mutant MALAT1 fragment in a Renilla plasmid using Lipofectamine 2000 (Invitrogen). Reporter gene assays were performed 48 h posttransfection using the Dual-Luciferase Assay System. Firefly luciferase activity was normalized to the corresponding Renilla luciferase activity to account for differences in transfection efficiency. All experiments were performed in duplicate and repeated at least 3 times.

### Cell viability and cell cycle analyses

Cell viability was analyzed using 3-(4, 5-Dimethylthiazol-2-yl)-2, 5-diphenyltetra- zolium bromide (MTT, Sigma) assays as previously described. Briefly, 5 × 10^3^ cells per well were seeded into a 96-well plate. After miRNA transfection, the cells were maintained for 72 hours and cell viabilities were determined using a Benchmark PlusTM microplate spectrometer (Bio-Rad). For cell cycle analysis, the cells were harvested 48 h following transfection, washed with PBS, and fixed in 75% ethanol at −20°C. After overnight fixation, the cells were washed with PBS and stained with propidium iodide (Beckman Coulter, Fullerton, CA) for 30 min. Cell cycle analysis was performed using the BD Flow Cytometry System with FACSDiva software (BD Biosciences, Franklin Lakes, USA). The cell cycle distribution is presented as the percentage of cells in G1, S and G2 phases. The data were analyzed with FlowJo v5.7.2.

### Xenograft tumor model

Both miR-124 expression and the MALAT1 expression vector were constructed and transfected with Lipofectamine 2000 reagent (Invitrogen). In total, 1 × 10^7^ breast cancer cells and their parallel control cells were subcutaneously injected into the same nude mice aged 4 weeks. The tumor cells were allowed to grow for 4 weeks. The tumor growth was evaluated by measurement of the length and the width with electronic calipers, and the tumor volume was calculated using the formula: Volume = (*Length × Width*^2^)/2. One month later, the nude mice were sacrificed and the tumor tissues were excised, weighed and fixed in 4% paraformaldehyde solution for further study. The tumor growth was evaluated by the value of tumor volume (mean ± SD), which was plotted against time. Animal handling was conducted in accordance with the approval of the Animal Care and Use Committee of Nanjing Medical University.

### Statistical analyses

The significance of differences between groups was estimated by Student's *t*-test. Mann–Whitney U test was used to compare differences between miR-124 expression and other characteristics. Cases were divided into two groups, high or low, using the median expression level of miR-124 as a cutoff. The Chi-square or Fisher's exact test was used to evaluate the relationship between miR-124 expression and clinical features. Survival analyses were performed using the Kaplan–Meier method and the log-rank test. Hazard ratios and 95% confidence intervals (CIs) were calculated using Cox proportional hazards model. *P* value < 0.05 was considered statistically significant. All statistical analyses were performed using the SPSS version 19.0 (SPSS Inc., IL, USA).

## SUPPLEMENTARY MATERIALS FIGURES AND TABLES


