# Supplementary material for: miR-124 downregulation leads to breast cancer progression via LncRNA-MALAT1 regulation and CDK4/E2F1 signal activation
Source: Oncotarget. 2016 Feb 22;7(13):16205–16. doi: 10.18632/oncotarget.7578 (PMC4941308; doi:10.18632/oncotarget.7578)
Supplement: Supplementary file 1 [file oncotarget-07-16205-s001.pdf]

miR-124 downregulation leads to breast cancer progression via LncRNA-MALAT1 regulation and CDK4/E2F1 signal activation

Supplementary Materials

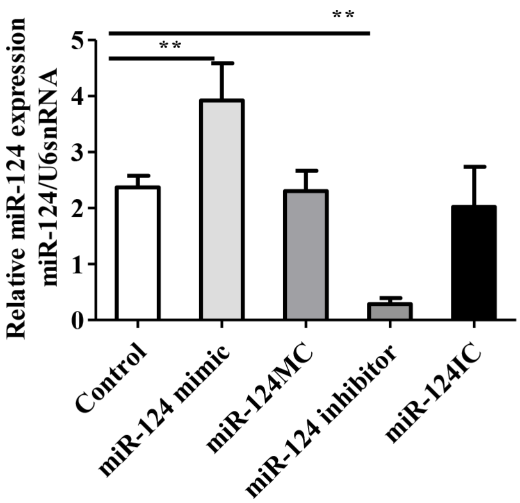

Supplementary Figure S1: The expression of miR-124 in breast cancer cells. MCF-7 cells with miR-124 mimic, miR-124 inhibitor treatment, and miR-124 expression was examined by qRT-PCR and normalized to U6snRNA expression in breast cancer cells. PBS as control. The data are shown as the mean ± SD from three independent experiments. *$p < 0.05$, **$p < 0.01$.

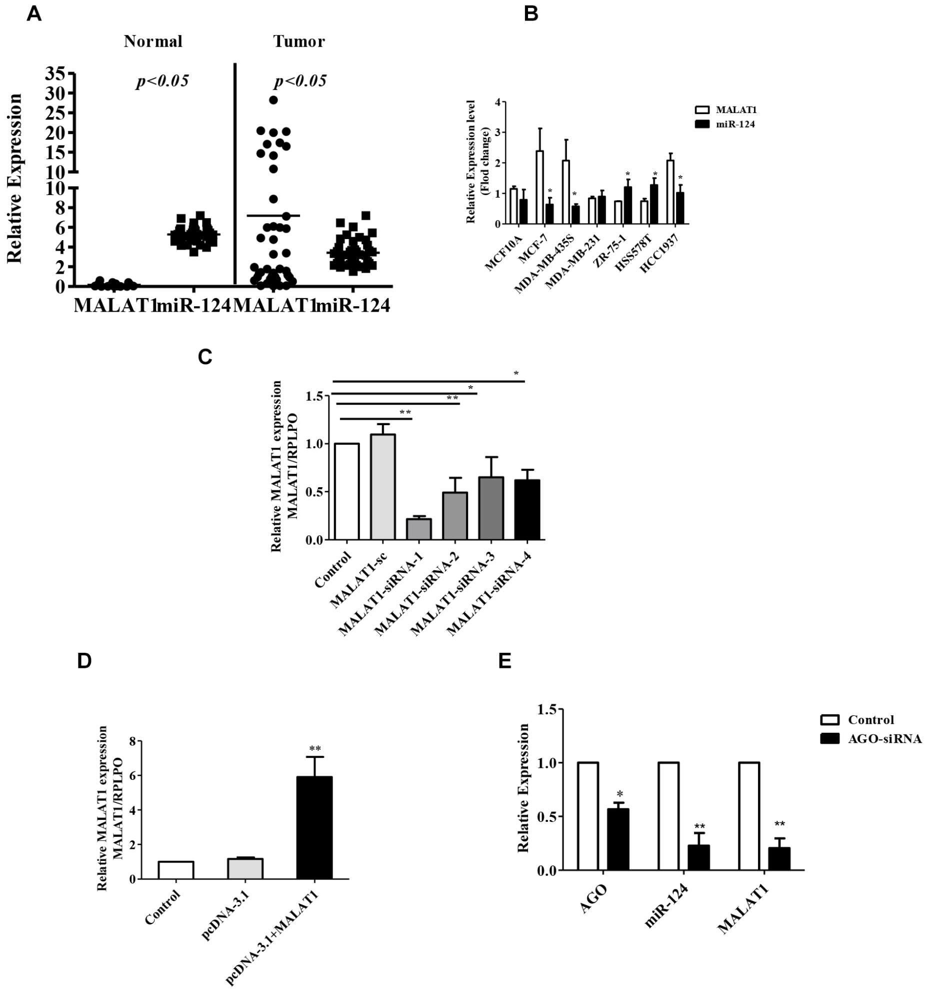

Supplementary Figure S2: The expression of MALAT1 and miR-124 in breast cancer tissues and cells. (**A**) MALAT1 expression and miR-124 expression were examined by qRT-PCR and normalized to RPLPO expression and U6snRNA expression in breast cancer tissues (Cancer) compared with adjacent non-cancerous tissues (Normal). (**B**) MALAT1 expression level and miR-124 were analyzed in different breast cancer cell lines by qRT-PCR. RPLPO and U6snRNA were treated as internal control. (**C**) MCF-7 cells with MALAT1-siRNA treatment, and MALAT1 expression was examined by qRT-PCR and normalized to RPLPO expression in breast cancer cells. MALAT1-sc as control. (**D**) MCF-7 cells with pcDNA-3.1+MALAT1 treatment, and MALAT1 expression was examined by qRT-PCR and normalized to RPLPO expression in breast cancer cells. pcDNA-3.1 was as control. (**E**) MCF-7 cells with AGO-siRNA treatment, and MALAT1 expression and miR-124 were examined by qRT-PCR in breast cancer cells. The data are shown as the mean ± SD from three independent experiments. *$p < 0.05$, **$p < 0.01$.

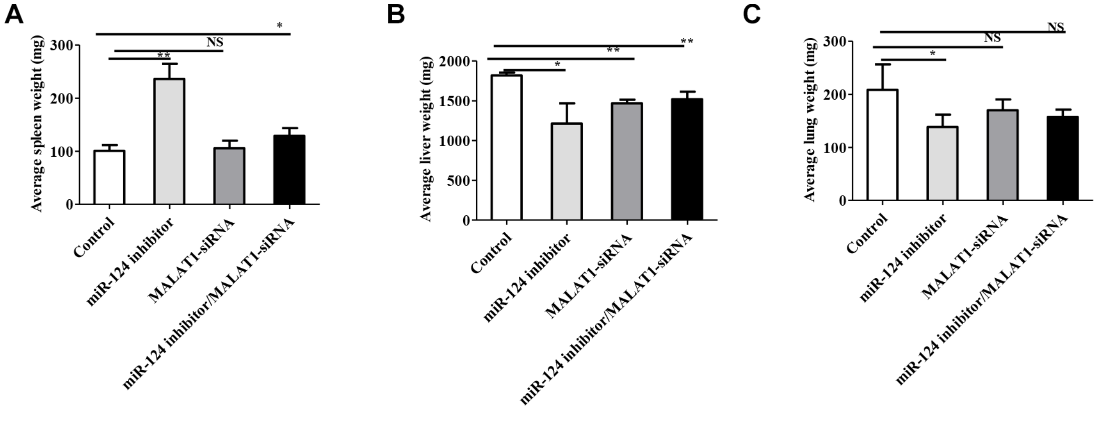

Supplementary Figure S3: MALAT1 regulates tumor growth through miR-124 *in vivo*. The spleen, liver and lung were determined when the mice was sacrificed. Derived from PBS as control ($n = 3$), miR-124 inhibitor ($n = 5$), MALAT1-siRNA ($n = 5$) and miR-124 inhibitor+MALAT1-siRNA ($n = 5$). The data are shown as the mean ± SD from three independent experiments with similar results *$p < 0.05$, **$p < 0.01$, *NS*, No significant difference.

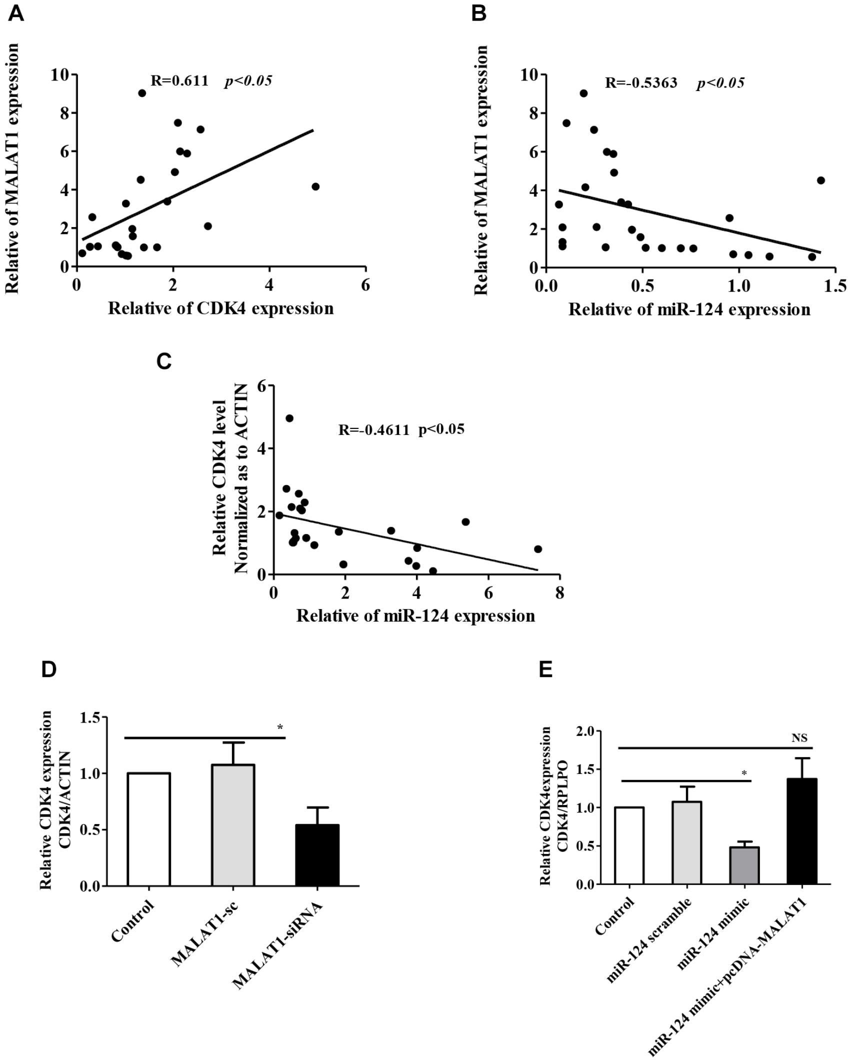

Supplementary Figure S4: MALAT1 regulates CDK4 expression, target of mIR-124. (**A**) The correlation between MALAT1 mRNA levels and CDK4 levels was examined by qRT-PCR in breast cancer tissues. (**B**) The correlation between MALAT1 mRNA levels and miR-124 levels was examined by qRT-PCR in breast cancer tissues. (**C**) The correlation between miR-124 levels and CDK4 levels was examined by qRT-PCR in breast cancer tissues. (**D**) Breast cancer cells with MALAT1-siRNA treatment, and CDK4 expression was examined by qRT-PCR in breast cancer cells. MALAT1-sc was as control. (**E**) MCF-7 cells with miR-124 scramble, miR-124 mimic and miR-124 mimic+pcDNA3.1+MALAT1 treatment and CDK4 expression was examined by qRT-PCR in breast cancer cells. The data are shown as the mean ± SD from three independent experiments. **p* < 0.05, ***p* < 0.01, *NS*, No significant difference.

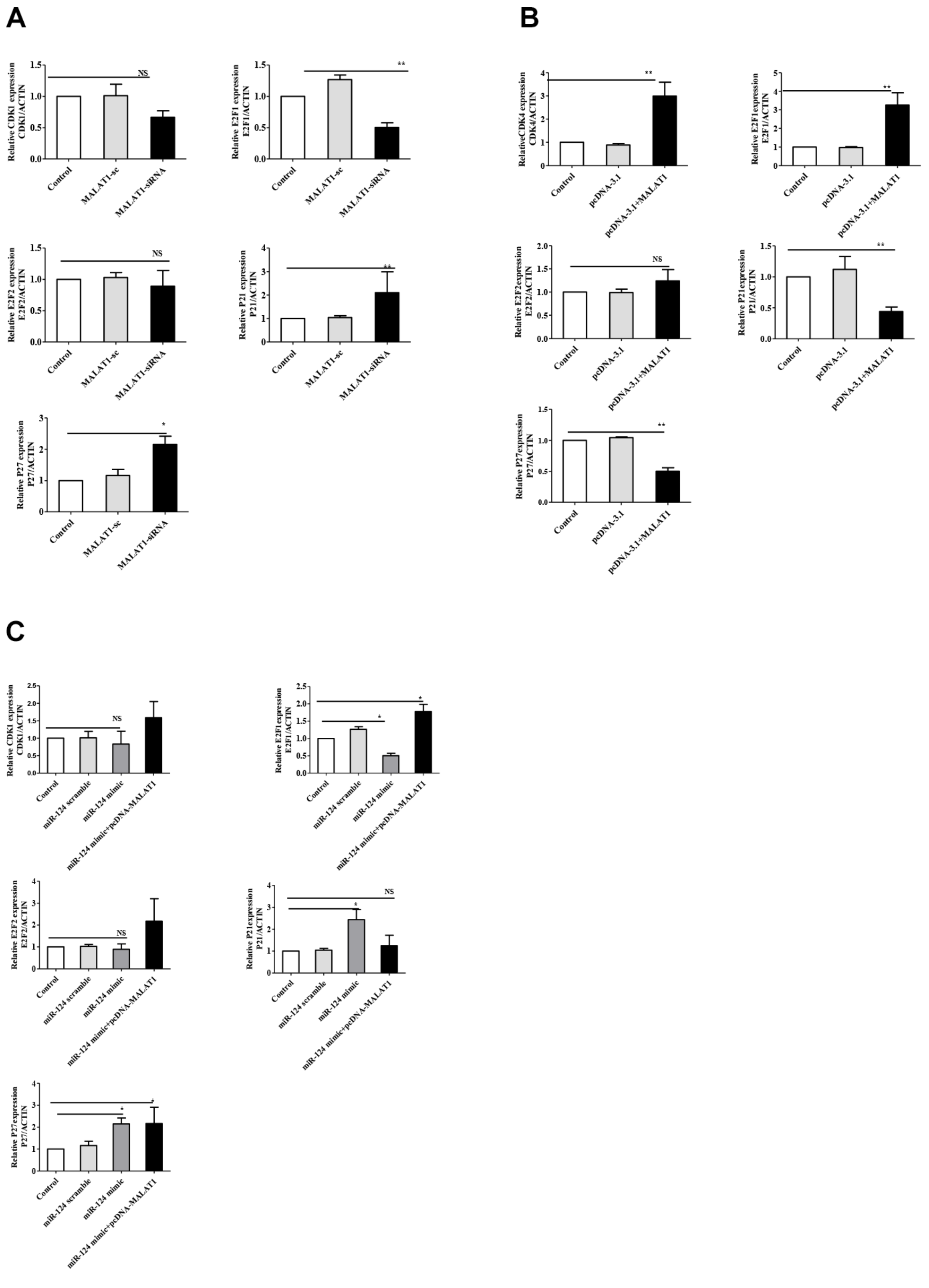

Supplementary Figure S5: MALAT1 induced cell proliferation through CDK4/E2F1 signaling pathway in breast cancer. (**A**) Breast cancer cells with MALAT1-siRNA treatment, and CDK1, E2F1, E2F2, P21, P27 expression were examined by qRT-PCR in breast cancer cells. MALAT1-sc as control. (**B**) Breast cancer cells with pcDNA-3.1+MALAT1 treatment, and CDK4, E2F1, E2F2, P21, P27 expression were examined by qRT-PCR in breast cancer cells. pcDNA-3.1 was as control. (**C**) Breast cancer cells with miR-124 scramble, miR-124 mimic and miR-124 mimic+pcDNA3.1+MALAT1 treatment, and CDK1, E2F1, E2F2, P21, P27 expression were examined by qRT-PCR in breast cancer cells. PBS as control. The data are shown as the mean ± SD from three independent experiments. *$p < 0.05$, **$p < 0.01$, *NS*, No significant difference.

Supplementary Table S1: The relationship between miR-124 expression and clinicopathologic parameters

Characteristics	miR-124 expression		*P* value
	Low (n = 24) No. (%)	High (n = 24) No. (%)	
Age(years)			0.042
≥ 50	17 (70.8)	10 (41.7)	
< 50	7 (29.2)	14 (58.3)	
CD44			0.743
Positive	17 (73.9)	16 (69.6)	
Negative	6 (26.1)	7 (30.4)	
COX-2			0.299
Positive	12 (54.5)	16 (69.6)	
Negative	10 (45.5)	7 (30.4)	
EGFR			0.655
Positive	4 (17.4)	2 (9.1)	
Negative	19 (82.6)	20 (90.9)	
ER			0.760
Positive	15 (65.2)	14 (60.9)	
Negative	8 (34.8)	9 (39.1)	
PR			1.000
Positive	14 (60.9)	14 (60.9)	
Negative	9 (39.1)	9 (39.1)	
HER-2			0.767
Positive	12 (52.2)	13 (56.5)	
Negative	11 (47.8)	10 (43.5)	
PGP			0.305
Positive	10 (43.5)	6 (28.6)	
Negative	13 (56.5)	15 (71.4)	
Lymphnode metastasis			0.562
Positive	12 (50)	10 (41.7)	
Negative	12 (50)	14 (58.3)	

EGFR epidermal growth factor receptor, ER Estrogen receptor, PR Progesterone receptor, HER2 human epidermal growth factor receptor 2, PGP P-glycoprotein.

Supplemental Table S2: Univariate and multivariate analysis of different prognostic factors for disease-free survival in patients with breast cancer

Prognostic factors *p* value	HR	95 % CI		*p* value	HR	95 % CI
Age (year ≥ 50 / ≤ 50)	0.846	0.442–1.619	0.613	0.784	0.318–1.931	.597
ER (negative/positive)	1.05	0.525–2.097	0.891	1.178	0.432–3.208	.749
HER2 (negative/positive)	1.139	0.569–2.279	0.713	1.503	0.354–6.374	.581
CD44 (negative/positive)	0.975	0.448–2.122	0.949	0.708	0.153–3.268	.658
COX2 (negative/positive)	1.495	0.722–3.093	0.278	1.388	0.597–3.228	.447
EGFR (positive/negative)	1.335	0.504–3.535	0.561	1.911	0.529–6.901	.323
PGP (negative/positive)	1.598	0.772–3.308	0.207	1.515	0.526–4.365	.441
Lymph node metastasis	0.704	0.365–1.359	0.296	0.674	0.144–3.151	.616
miR-124 (low/high)	0.753	0.388–1.460	0.0401	0.506	0.201–1.274	.0148

Supplemental Table S3: Oligonucleotide sequence of PCR primers

Name	Sequences(5′–3′)
Primers for real-time quantitative RT-PCR	
CDK1-F	GGATGTGCTTATGCAGGATTCC
CDK1-R	CATGTACTGACCAGGAGGGATAG
E2F1-F	ACGTGACGTGTCAGGACCT
E2F1-R	GATCGGGCCTTGTTTGCTCTT
E2F2-F	CCGACAGGACTGAGGACAAC
E2F2-R	GCACAGGTAGACTTCGATGGG
P21-F	GCAGACCCAGCATGACAGATTT
P21-R	GGATTAGGGCTTCCTCTTGGA
P27-F	ATCACAAACCCCTAGAGGGCA
P27-R	GGGTCTGTAGTAGAACTCGGG
CDK4-F	TCGTGAGGTGGCTTTACTGAGGC
CDK4-R	CTTAGGTCCTGGTCTACATGCTC
MALAT1-F	GGTAACGATGG TGTCGAGGTC
MALAT1-R	CCAGCATTACAGTTCTTGAACATG
MALAT1-01-F	CCTCCTGATATGCAAAGTTTG
MALAT1-01-R	GGCACGGCAGAAGCAGAGAGC
MALAT1-201-F	CCTCCTGATATGCAAAGTTT
MALAT1-201-R	TGGCACGGCAGAAGCAGAGAGC
ACTIN-F	TACAATGAGCTGCGTGTGGCTCC
ACTIN-R	CTGGATAGCAACGTACATGGCTG
GAPDH-F	TGTGTCCGTCGTGGATCTGA
GAPDH-R	CCTGCTTCACCACCTTCTTGA
RPLPO-F	TCGACAATGGCAGCATCTAC
RPLPO-R	ATCCGTCTCCACAGACAAGG
miR-124-RT	GTCGTATCCAGTGCAGGGTCCGAGGTATT CGCACTGGATACGACGGCATTCT
miR-124-F	GATACTCATAAGGCACGCGG
miR-124-R	GTGCAGGGTCCGAGGT
miR-124-F1	TCCGTGTTCACAGCGGAC
miR-124-R2	CATTCACCGCGTGCCTTA
U6SnRNA-F	GTGCTCGCTTCGGCAGCACATA
U6SnRNA-R	GGAACGCTTCACGAATTTGCGTGTC

Primers for CDK4 expression vector

CDK4-F CGGAATTCCACCTCCTGTCCGCCCCTCA (EcoR1)
CDK4-R CGGGATCCCTCCGGATTACCTTCATCCT (BAMH1)
miR-124-F CCCAAGCTTATTCCATCTTCTACCCACCC (HindIII)
miR-124-R CCGCTCGAGTTTGCATCTCTAAGCCCCTG (Xho1)
MALAT1-F1 CGAGCTCTTTTCTTGCTCACATCTCTC (SacI)
MALAT1-R1CCCAAGCTTCTTTGTTCTTTTAGGCAGGC (HindIII)
MALAT1-F2 GCCAAGCTTGATGGAGTTTTCCTCTTGTTGCCCA (HindIII)
MALAT1-R2 GCGCTCGAGTAGGCAGGCTTGTTCCCTGACA (Xho1)

Primers for RNA Oligonucleotides

miR-124 mimic	UAAGGCACGCGGUGAAUGCC<br>CAUUCACCGCGUGCCUUAUU
miR-124 inhibitor	GGCAUUCACCGCGUGCCUUA
negative control	UUCUCCGAACGUGUCACGUTT
miRNA inhibitor negative control	CAGUACUUUUGUGUAGUACAA
si-scramble	ACGUGACACGUUCGGAGAAdtdt
siAGO	UUCAGAUGGACUUCCGUGCdtdt
siCDK4	CCAGGACCUAAGGACAUAU
siCtrl	CGUACGCGGAAUACUUCGAdTdT<br>UCGAAGUAUUCCGCGUACG
siMALAT1-1	GAGCAAAGGAAGUGGCUUA<br>UAAGCCACUUCCUUUGCUC
siMALAT1-2	GCGGAAGCUGAUCUCCAAU<br>AUUGGAGAUCAGCUUCCGC
siMALAT1-3	GGAAGUAAUUCAAGAUCAA<br>UUGAUCUUGAAUUACUUCC
siMALAT1-4	GCAAAUGAAAGCUACCAAU<br>AUUGGUAGCUUUCAUUUGC
